# Childhood disability in Aboriginal and Torres Strait Islander peoples: a literature review

**DOI:** 10.1186/1475-9276-12-7

**Published:** 2013-01-18

**Authors:** Michelle DiGiacomo, Patricia M Davidson, Penelope Abbott, Patricia Delaney, Tessa Dharmendra, Sarah J McGrath, Joanne Delaney, Frank Vincent

**Affiliations:** 1 Centre for Cardiovascular and Chronic Care; Faculty of Health, University of Technology Sydney, PO Box 123, Broadway, NSW 2007, Australia; 2 Curtin University, Curtin Health Innovation Research Institute, Perth 6845, Australia; 3 Aboriginal Medical Service Western Sydney, PO Box 3160, Mt Druitt, NSW 2770, Australia; 4 University of Western Sydney, Penrith, New South Wales 1797, Australia

**Keywords:** Aboriginal and Torres strait islander, Childhood, Disability, Early intervention

## Abstract

**Introduction:**

Aboriginal and Torres Strait Islander children have higher rates of disability than non-Indigenous children and are considered doubly disadvantaged, yet there is very little data reflecting prevalence and service access to inform design and delivery of services. Failing to address physical, social, and psychological factors can have life-long consequences and perpetuate longstanding health disparities.

**Methods:**

A narrative literature review was undertaken to identify peer reviewed literature describing factors impacting on the prevention, recognition, and access to support and management of disability in Indigenous Australian children.

**Results:**

Twenty-seven peer-reviewed journal articles met inclusion criteria. The majority of articles focused on the hearing loss and learning disabilities consequent of otitis media. Few articles reported data on urban or metropolitan Indigenous populations or described interventions. Individual/community-, provider-, and systems level factors were identified as impacting on recognition and management of disability in young Indigenous children.

**Conclusions:**

Given the burden of childhood disability, the limited literature retrieved is concerning as this is a barometer of activity and investment. Solutions addressing childhood disability will require collaboration between health, social and educational disciplines as well as an increased investment in prevention, identification and promotion of access.

## Introduction

Early childhood is a critical period that can influence a range of health and social outcomes throughout the life course. In Australia, an economically prosperous and generally healthy nation, Aboriginal and Torres Strait Islander Peoples (hereafter, Indigenous) suffer worse health, disadvantage, and disability comparable to many developing nations [1]. Dispossession, disempowerment, and racism contribute to adverse outcomes [2,3]. Indigenous children with a disability are considered ‘doubly disadvantaged’, and failing to address physical, social and psychological factors can have life-long consequences [4,5]. High rates of childhood disability in Indigenous children fuel health inequities and should be an important focus of attention for policy makers and health professionals.

### Disability in Indigenous Australians

For the purposes of this paper, the term disability refers to the long-term physical, mental, intellectual, or sensory impairments that hinder full and effective participation in society on an equal basis with others [6]. Rates of disability in Indigenous adults and children are higher than in non-Indigenous Australians. Indigenous adults are 1½ times more likely than non-Indigenous Australians to have a disability or long-term health condition, more than 3 times as likely to have an intellectual disability, and more than twice as likely to require support meeting self-care, communication or mobility needs [7,8]. Similarly, Indigenous children are 30% more likely to require assistance with learning or communicating, have a core activity need for assistance, and a profound or severe disability, than age-matched non-Indigenous children [3]. Up to 27% of Indigenous children in Western Australia (WA) have limitations in vision, hearing, or speech [9]; a statistic likely reflecting Australia having one of the highest rates of otitis media (OM) in the world [10,11]. Other studies have identified lower scores on performance and development in young Indigenous children as compared to non-Indigenous children [12,13].

Despite these disparities, data on prevalence, type, and service access among Indigenous children in Australia is limited, particularly in urban populations [11,14,15]. Only recently has the Australian national census sought information on disability for Indigenous individuals under the age of fifteen [3]. Problems with Indigenous identification, high non-response rates, and different conceptualisations of disability may mask hidden disability and contribute to under-reporting [14,16].

### Low service use

In addition to little prevalence data, which precludes discussion of service access in children, it has been reported that few Indigenous people with a disability access services. In 2008–09, only 5% of users of specialised support services were Indigenous [17], indicating that they are not participating in available programs [18]. In New South Wales, which has the largest population of Indigenous people in Australia, and the largest population with severe or profound core activity limitations, rates of service provision to Indigenous people are below the national average [17]. Barriers in accessing support have been attributed to remoteness, social marginalisation, cultural attitudes towards disability, and culturally inappropriate services [19].

Although not directly targeting disability, cost effective quality early interventions, such as pre-school, have been reported to be key points where development indicators can trigger support, potentially making a difference to life-long outcomes [20,21]. In Australia, just 25% of Indigenous children aged 3.5-4.5 attend any formal early childhood services and these children are least likely to have access to high quality pre-school education [22,23].

### Consequences of lack of support

This lack of support and therapies in the early years of life can have devastating consequences for the child, their parents/carers, family, and community; contributing to cycles of disadvantage. Investment in the early years through policies that address social determinants of health has potential to reduce social, economic, and health disparities within a generation [2,24]. The need to inform such policies through measuring and understanding the problem and assessing the impact of action has driven this review. Specifically, we aimed to answer the following question: What are the factors impacting on the prevention, recognition, and access to support and management of disability in Indigenous Australian children? To answer this question, we conducted an integrative narrative review to ascertain the state of the science, potential solutions, and gaps in services and research.

## Methods

### Data collection

We undertook an integrative review which is a method that incorporates disparate sources, methods, and types of literature and facilitates development of comprehensive accounts of phenomena [25] while summarising literature, identifying gaps, and recommending further research in a given area [26]. In contrast to a systematic review, an integrative review, although conducted with systematic methods, discusses and summarizes literature on a particular topic, without generating a pooled summary or focusing on questions of efficacy.

The literature search strategy was designed and conducted in consultation with a health librarian in August 2011. Peer-reviewed literature was identified via searches of Medline, PsychInfo, Cumulative Index to Nursing and Allied Health Literature (CINAHL), Indigenous Australia, Australian Public Affairs Information Service-Aboriginal and Torres Strait Islanders (APAIS-ATSIS), Aboriginal and Torres Strait Islanders Health (ATSIHealth), the Australian Indigenous Health InfoNet, Education Resources Information Center (ERIC), and Cochrane Library databases and the Google Scholar search engine. Database searches were limited to articles written in English and published between 1990 – July 2011. Additional literature was identified via retrieved studies’ reference lists. Literature was included if it 1) centred on Indigenous Australian children or families; 2) centred on issues surrounding Indigenous Australian children with a disability or impairment; and 3) depicted aspects of prevention or recognition of disability or access to support, treatment, or management. Articles were excluded if they did not report separately on Australian Aboriginal and Torres Strait Islander Peoples or did not concern childhood disability. Search terms were Medical Subject Headings (MeSH) terms and keywords including derivations of child, (newborn, baby, babies, infant, infants, children, childhood) and Indigenous (Indigenous, Australian Indigenous, Australian Indigenous, Aborigine, Oceanic ancestry group) and disability (intellectual disability, learning disorder, language disorder, communication disorder, hearing impaired, hearing loss, hearing disorder, visually impaired, mentally disabled, developmental disability, attention deficit, disruptive behavior disorders, child behavior disorders, child development disorders, motor skills disorders, cognition disorder, speech, language disorders, autism, autistic disorder, Aspberger syndrome, physical disability) tailored to relevant databases. For the inclusive purposes of this review, the term disability encompassed developmental delay, intellectual disability, sensory impairment, and physical disability. Theoretical and empirical papers were included and no restrictions were imposed on study design or methodology.

The grey literature was searched using broad terms depicting Indigenous children with disability after identifying websites of relevant government and non-government organizations in Australia, as well as Australian universities and research centres. We contacted key authors to request copies of relevant reports not available via the internet. Additional grey literature was sourced using the databases listed above, the internet search engine Google, and via hand searching of reference lists.

### Data evaluation and analysis

Two authors independently reviewed all titles and abstracts for relevance to the review topic. They obtained full copies of retained articles for detailed examination to determine suitability for inclusion. Any disagreement between authors was resolved by a third author. Two authors extracted data from retrieved documents, into a customised data extraction spreadsheet with columns depicting disability focus, methodological and intervention features (if available), aims, key findings, and information pertaining to prevention, recognition, and access to support and management of disability. Strength of findings of primary sources were evaluated via MeInyk and Fineout-Overholt’s Levels of Evidence ranking system [27]. In addition, observational studies were evaluated using the STrengthening the Reporting of OBservational studies in Epidemiology checklist (STROBE) checklist [28], and intervention studies were appraised via the Transparent Reporting of Evaluations with Nonrandomized Designs (TREND) checklist for non-randomised health and social interventions [29]. Analysis proceeded with categorisation of data guided by the research question and the Chronic Care framework, which summarizes elements for improving care in health systems at individual, community, practice and organization levels [30].

## Results

**Figure 1:**
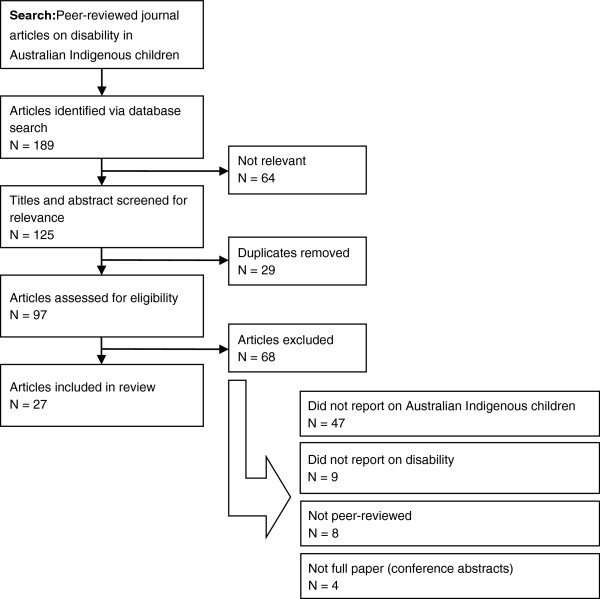
Literature selection process.

The literature search resulted in 189 peer-reviewed articles reporting both empirical studies and theoretical articles. Following application of inclusion and exclusion criteria, twenty-seven articles were included in the review (Figure 1). The majority of peer-reviewed articles (n = 20) described otitis media (OM) and hearing loss in Indigenous peoples. With the exception of studies describing national data, just four articles reported data on urban or metropolitan Indigenous populations. Nearly twenty per cent of the articles (n = 6) were over ten years old. The majority of articles reported on descriptive and observational studies (n = 11) (Table 1). STROBE scores ranged from 4 (indicating provision of little detail on which to judge veracity of findings) to 21 (indicating provision of detailed information) [28]. The mean STROBE score was 14.7 out of a possible 22 (67%).

**Table 1:** Observational studies

**First Author (year)**	**Disability/ impairment**	**Design**	**Level of Evidence**	**STROBE score**	**Population**	**Setting**	**Methods**	**Aims**	**Key Contributions**	**Conclusions/recommendations**	**Strengths**	**Weaknesses**	**Category**
Aithal, S. (2008)	Hearing	Observational	IV	17.5	Indigenous children (n = 15): 1) English- speaking w/no hearing loss; 2) learning ESL w/no hearing loss; 3) ESL with hearing loss; mean age 13	Island 80km north of Darwin	Hearing test	To examine the effect of hearing loss and native-language phonology on learning English by Australian Indigenous children	ESL children has slower recognition time of English words; hearing impairment related to OM made it even more difficult for ESL children	Phonological awareness programs need to be part of a reading program from preschool for ESL Indigenous children	Inclusion of control group	Sampling not described; use of interpreter not discussed	R
Gunasekera,H. (2007)	Hearing	Cluster survey of consecutive primary health consultations	IV	21	Primary care consults on OM in Indigenous (n = 280) and non- Indigenous (n = 8,510) aged 0–18 years	Australia	secondary analysis of national survey	To assess clinical management of OM in Aboriginal children	Indigenous kids more likely to have severe OM, but not more likely to receive oral antibiotics, ear syringing, referral to specialist.	Indigenous children are 5 times more likely to be diagnosed with severe OM than non-indigenous children, but management is not substantially different; inconsistent with established national guidelines.	Representative national survey of health care consultations; randomized sampling of health consultations	Prevalence and incidence infeasible; no data on progress, treatment of cases; age range only 0-18	A
Gunasekera,H. (2009)	Hearing	Cross-sectional survey	VI	18	AMS medical practitioners managing children's OM in December 2006	Australia	Postal survey	To compare the burden of OM managed by AMS practitioners and the availability of specialist ear health services in rural/remote versus urban Australian settings	More cases managed/week in rural remote and more reported relevant services were not available locally; audiology waiting times longer than the recommended 3 months; equal proportions of urban/rural reported ENT waiting time longer than the recommended 6 months	Need for adequate funding of visiting services in rural/remote settings and outreach programs delivered by Aboriginal HPs, increasing frequency of audiologist visits to rural and remote locations, proportionate hearing service expenditure should reflect the population’s need as well as rurality indices.	Nearly 3/4 of AMS’s represented; audiology assessments had face validity with clinicians an in line with international	55% response rate; no independently verification of waiting times; no comparison	A
Howard, D. (1991)	Hearing	mixed-method; ethnography	VI	8.5	23 Aboriginal students in multi- grade Aboriginal class	Darwin	observation, physiological assessment, teacher interviews & survey	To investigate relationship between Aboriginal children's hearing loss and their learning.	30% of children had hearing loss; teacher- oriented learning behavior associated with attendance and achievement	Hearing loss appears to magnify difficulties in cross-cultural education; need to consider how schools currently meet needs of Aboriginal students with hearing loss and how Western schooling can be altered to better meet these needs.	integration of interview and survey data; Aboriginal teaching staff included; observation longitudinal	limited information on recruitment, interviews; single observer	R
Howard, D. (2006)	Hearing	Case study	VI	14.5	Urban Aboriginal children	Darwin	classroom observation and hearing assessment	To identify cultural differences in attentiveness between Aboriginal and non- Aboriginal children; to examine differences between urban hearing and hearing-impaired Aboriginal children.	Cultural differences in attentiveness style in Aboriginal students with and without hearing loss may lead to inaccurate assessments by assessors.	Without formal screening, cross-cultural misunderstanding is likely to inhibit appropriate teacher referrals of Aboriginal children for hearing tests; regular school hearing screening for Aboriginal children is needed; Teachers to be aware of possible behavioral indicators of Aboriginal children’s hearing loss.	mixed method; integrated data	small sample; single observer	R
D'Aprano, A. (2011)	Development	tool assessment via cross-sectional screening	VI	18	124 Australian Aboriginal children, aged 3–7 years	3 remote communities in NT	pediatrician screening using standardized tool	To trial the Brigance developmental screening tool to identify Australian Aboriginal children at risk of developmental disability and requiring assessment.	All children scored below the cut-off for likely having developmental disabilities or academic delays; all well behind their age peers	Language and cultural relevance, and the method of administration limit the use of tool; need to adapt appropriate instrument to guide developmental surveillance and monitoring in remote Australian Aboriginal communities that incorporates families	Aboriginal research staff involvement; tool sensitive to psychosocial disadvantage	remote-only; recruitment not described; language barriers for some participants; cultural relevance not established for some	R
Howard, D. (2004)	Hearing	Cross-sectional survey	VI	14	167 Aboriginal children	Remote NT	teacher survey and child hearing assessment	To asses extent of social and educational difficulties across cross cultural classrooms.	Aboriginal children with bilateral hearing loss participate less in class, are more disruptive, and require more one to one assistance	Ensure access to Aboriginal teachers and tutors; train Aboriginal teachers and tutors in issues around hearing loss; provide professional development to non-Aboriginal teachers.	teacher report integrated with objective tests; Aboriginal teaching staff included	remote-only; definitive conclusions implausible; no sample size or sampling frame information	R-Q
Bennett, B. (2010)	Development	tool validation within prospective longitudinal cohort study	IV	20	55 urban Aboriginal children at 12 months of age	Southwest Sydney	structured and semi-structured questionnaire; interview; physical exam; social, motor, hearing and speech, eye hand coordination, and reasoning	To determine appropriateness of Griffiths’ Mental Developmental Scales to assess development in cohort of urban Aboriginal children	No significant differences except Gudaga performance scores were significantly less than the reasoning scores in the Griffiths’ standardization sample	Griffiths’ Mental Developmental Scales may be appropriate for urban Aboriginal infants.	Questionnaire administration in person; physician report; 100% response rate; prospective	small sample frame; reasons for poor reasoning performance unclear; definitive conclusions cannot be drawn	R, S
Aithal, V. (2006)	Hearing	Observational; Cross- sectional hearing test; comparison group	VI	14.5	36 Aboriginal children from Tiwi Islands with OM and some hearing loss; (mean age 10); Control group - 62 children from Darwin (normal hearing; mean age 13)	Island 80km north of Darwin and Darwin	Hearing test	To assess utility of masking level difference (MLD) as a measure to detect hearing loss in Aboriginal students with OM history	Aboriginal children showed lower MLD than control group. Auditory processing disorders (APDs) related to early auditory deprivation may have significant adverse effects on school performance.	MLD a less culturally biased measure and more easily administered than many speech and language test procedures.	Comparison group, use of MLD	Sampling not described; uneven groups	S
Nelson, A (2007, 2004)	General disability	Mixed-method (qualitative/quantitative)	VI	12.5	Urban Indigenous families & OTs	Brisbane	Interviews, focus groups, questionnaires	To investigate what constitutes a socially and culturally appropriate OT service for urban Indigenous Australian families in Brisbane	Service provision in context of school favorably; need to develop effective relationships and qualities; understand different backgrounds of client and therapist; address logistical issues of service delivery	OTs may need to make changes to the way in which they organize and deliver services to Indigenous clients.	parents included in sample; majority of participants Indigenous; service coordinated by Aboriginal health service; multiple informants and methods; facilitated reflection of service and practice	limited to 1 service type; children's perspectives not included; potential for response bias	S
Partington, G. (2006)	Hearing	Observational	VI	4	>500 Indigenous students preschool - year 3 (ages 5–8) from 16 schools, >80 teachers and assistants	Western Australia	multi-modal observation, evaluation spanning 2 years; teacher training and interviews, ear health assessment, achievement records, data mapping of classroom observations	To outline effective teaching strategies to improve literacy and education outcomes of Indigenous students	A variety of teaching strategies and environment likely to assist in improving educational outcomes	Teachers and their schools were important factors in improving educational outcomes	theoretical sampling of successful teachers enabled focus on key characteristics, practices	potential for social desirability; no description of sampling, recruitment, response rate; theoretical sampling potentially narrow	S, I

English as a second language (ESL); Otitis Media (OM); Aboriginal Medical Service (AMS); Ears, nose, and throat (ENT); masking level difference (MLD); Auditory processing disorders (APDs); Occupational therapists (OTs); kilometer (km); Category: (R-recognition/awareness; A-Access; S-Solutions; I-Intervention; Q-Sequelae/outcomes); Level of Evidence: I Evidence from a systematic review, meta-analysis of all relevant randomized control trials (RCT) (Strongest); II Evidence from at least one well-designed RCT; III Evidence from well-designed controlled trials without randomization; IV Evidence from well-designed case–control and cohort studies; V Evidence from systematic reviews of descriptive and qualitative studies; VI Evidence from single descriptive or qualitative study; VII Evidence from the opinion of authorities or expert committee reports (Weakest).

Just four intervention studies were identified and these addressed stigma, sound amplification (management), and screening (recognition) (Table 2). All reported beneficial outcomes, three of which involved teaching strategies and/or hearing support in classrooms with evaluations of literacy, communication, and other educational outcomes. The mean TREND score was 11.9 out of a possible 22 (54%).

**Table 2:** Intervention studies

**First Author (year)**	**Disability/ impairment**	**Design**	**Aims**	**Level of Evidence**	**TREND Scores**	**Population**	**Geographic Setting**	**Intervention type**	**Methods**	**Indigenous involvement**	**Recruitment**	**Control group**	**Language**	**Content/components**	**Duration**	**Evaluation**	**Key Findings**	**Conclusions/recommendations**	**Category**
Ryan, B. (2006)	Hearing	Experiment; intervention	To investigate Indigenous Australian children's attitudes of peers wearing hearing aids	VI	13	60 Indigenous Australian children aged 5–12 years (mean age 9)	3 urban schools in Alice Springs	learning- based desensitization program	Experiment (photographs of Aboriginal people with and without hearing aids, attitudes surveyed, 20 min. educational intervention on benefits of hearing aids)	Community Consult	No info	Reverse ordered	English and Tiwi	discussion-based intervention was designed to encourage the participants to reduce stigma and negative attitudes towards people who wear hearing aids; demonstrations; An audio example; scenarios	10 minutes	Survey	Children had negative attitudes towards others with hearing aids; intervention had significant effect on attitudes	Children had more negative attitude towards peers with hearing aids; intervention had significant effect on attitudes; Potential for negative attitudes towards peers w/hearing aids to be changed via learning-based discussion aimed at reducing negative attitudes.	A
Strange, A. (2008)	Hearing	Experimental; intervention	Identify the negative stigmas attached to hearing aids, increase awareness of attitudes	VI	13	62 Indigenous adolescents boarding at high schools Alice Springs aged 12–18 (mean age 14)	Alice Springs	learning- based desensitization program	Experiment (photographs of Aboriginal people with and without hearing aids, attitudes surveyed, 20 min. educational intervention on benefits of hearing aids)	Community Consult	No info	Reverse ordered	English and Tiwi	discussion-based intervention was designed to encourage the participants to reduce stigma and negative attitudes towards people who wear hearing aids; demonstrations; An audio example; scenarios	20-30 minutes	Survey	greater visibility of the hearing aid, is associated with more negative attitudes by adolescents; intervention demonstrated some reduced stigma	Stigma and negative attitudes contribute to the low use of hearing aids in children; Need to develop appropriate strategies to decrease stigma and increase the use of amplification; appropriate attitude changing techniques interventions needed	A
Yonovitz, L. (2000)	Hearing	Intervention	Demonstrate link between hearing loss and low English literacy	VI	10.75	1,032 Indigenous students 4–22 years old representing 106 rural and remote communities	NT (Darwin and Alice Springs)	phonological awareness	Teacher in-service sessions; pre/post PA- EFL criterion- referenced, diagnostic tests	Not reported	No info	None	English	1)two-day workshops for each school for teachers and assistant; 2)provision of amplification systems and hearing aids; 3) ear, hearing, phonological awareness assessment; 4) reading, spelling assessment; (over 1 school year)	12 months	ear, hearing, phonological awareness, reading, and spelling assessments	Teacher training, hearing support services, screening, and phonological awareness intervention documented strong improvements in literacy and contributed to understanding relationship between ear disease and low literacy.	This intervention represents a feasible, adaptive program that can be used in combination with existing ESL curricula and should not cause interference with already published phonics programs.	S, I
Massie, R. (2004)	Hearing	Intervention	Identify effects of sound-field amplification on communication in classrooms of Aboriginal and Torres Strait Islander children	VI	11	64 Aboriginal and Torres Strait Islander students from 4 classrooms in two rural QLD schools	Rural QLD communities	amplification trial	Classroom observation; teacher questionnaire; assessments, modified Environmental Communication Profile, Screening Identification for Targeting Educational Risk rating scale	None reported	No info	None	English	amplification on/off conditions changed fortnightly	8 weeks	Teacher survey, sensory assessments	Sound-field amplification intervention encouraged the children to interact with teachers and peers in a proactive way.	No clear or enforceable standards for classroom acoustics in Australia exist; amplification may provide rapid, cost effective part of solution to improving the classroom listening environment.	S, I

Queensland (QLD); Category: (R-recognition/awareness; A-Access; S-Solutions; I-Intervention; Q-Sequelae/outcomes); Level of Evidence: I Evidence from a systematic review, meta-analysis of all relevant randomized control trials (RCT) (Strongest); II Evidence from at least one well-designed RCT; III Evidence from well-designed controlled trials without randomization; IV Evidence from well-designed case–control and cohort studies; V Evidence from systematic reviews of descriptive and qualitative studies; VI Evidence from single descriptive or qualitative study; VII Evidence from the opinion of authorities or expert committee reports (Weakest).

Five discussion papers (Table 3) and six literature reviews were identified, yet just one provided a detailed description of the review process (Table 4). Three reviews centred on hearing impairment and OM exclusively (Table 4). We identified six reports from the grey literature (Table 5). Although we endeavoured to ascertain factors impacting on prevention, recognition, and access to support and management of disability, we did not identify articles describing prevention other than a call for collaborative development of health-related promotional material to minimise ear infections [31]. Following this summative account of literature identified, we now provide a narrative summary using the framework of the Chronic Care model’s elements for improving care in health systems at multiple levels.

**Table 3:** Discussion papers

**First Author (year)**	**Disability/ impairment**	**Design**	**Level of Evidence**	**Population**	**Setting**	**Aims**	**Key Findings**	**Conclusions/recommendations**	**Category**
de Plevitz, L. (2006)	General Disability	Discussion paper; policy analysis	VII	Antidiscrimination law versus criteria for Indigenous students sent to special schooling	Australia	To argue that criteria developed for the allocation to special schooling may constitute indirect racial discrimination against Indigenous students	Education authorities could be liable despite unintentional effects; need for class allocation assessment by Indigenous educators.	National standards could be developed against which the reasons for placing students in special classes could be tested for their reliance on embedded cultural expectations and assumptions; need for the collection of national data on special schooling.	A
Gilroy, J. (2010)	General Disability	Policy analysis	VI	Policy documents published 1985- 2010	Australia	To analyze how New South Wales government-administered disability services positions and represents Aboriginal people with disability	Aboriginal people with disability were specialized field within the mainstream service system - 'cultural difference', 'remoteness' and 'vulnerability', but never a political group	The concept Aboriginal people with disability is a 'label' that conceptualizes what is not a normal person with a disability.	A
Cornish, D. (2011)	Hearing	Discussion paper	VII	Aboriginal children	Australia	To discuss link between hearing disability in Aboriginal children, language acquisition, and school performance	Reference to 2010 federal Senate inquiry into Indigenous ear health	Need to improve classroom acoustics in existing schools; police, courts and prisons provide more support for the hearing impaired	Q
Howard, D. (1992)	Hearing	Discussion paper/Tool	VII	Aboriginal children	Australia	To describe a hearing assessment feasible for school use.	Identification of Aboriginal children's hearing loss is important because of the major educational and social consequences of conductive hearing loss.	Aboriginal children's hearing loss is often not identified, in part because of 'masking' due to cultural differences. Simple speech reception game is effective in identifying children with hearing loss (for use by parents and teachers).	R, S
Henderson, I. (1993)	Hearing	observation; personal communication	VI	Aboriginal communities	North QLD & WA Aboriginal communities	To explore mismatch between remote Aboriginal concepts of disability and urban non-Aboriginal institutions	Remote area Aboriginal people have unique concepts of disability, hearing loss and otitis media	Consideration of variations in conceptualizations of disability is necessary in developing solutions	R

**Table 4:** Literature reviews

**First Author (year)**	**Disability/ impairment**	**Method description**	**Population**	**Setting**	**Aims**	**Key Findings**	**Conclusions/recommendations**	**Category**
Thorley, M. (2011)	Intellectual disability; general	No	Primary school- aged Indigenous Australian children	Australia	To present OTs with practice guidelines for conducting assessments with this population	Casual discussion with the client is preferable to a formal initial interview; Having Indigenous health worker present is appropriate and recommended	There is a general lack of research on assessments for Indigenous children; When working with Indigenous children: time should be invested in establishing relationship; familiar environment enabling therapist to build rapport; communication strategies including softer voice, avoiding jargon, using demonstration for unfamiliar tasks, using non-verbal media.	A, S
Williams, C. (2009)	Hearing	No	Indigenous and non-indigenous children	Australia	To discuss increased risk of negative cognitive and educational sequelae in Indigenous children with OM	Indigenous children may be at higher risk of cognitive and educational sequelae; early onset, more frequent infections, and infections of longer duration shown to be risk factors for long-term consequences.	Need for approaches to otitis media in Indigenous population that encompass both medical and educational considerations.	Q
Tourky, A. (1992)	Vision	No	Aboriginal children	Remote and isolated communities	To analyze the significance and the role played by the teachers in educating the Aboriginal children with visual impairments	Children with mild, moderate or severe visual problems develop behaviors (limited persistence due to fatigue, frustration with task completion or refusal to commence tasks, decreased motivation) that have negative effect on learning and school and non-school tasks.	Behaviors due to the visual problems have extremely significant negative impact on learning; measures needed to reduce adverse impacts.	Q
O'Neil, M. (2004)	General disability	no description of search terms, timeframe	Australian literature	Australia	To identify unique issues confronting Indigenous people with disabilities, their families, & communities	Indigenous people with a disability are generally not excluded from or stigmatized in their communities; disability may be viewed as a family or community problem, rather than a personal one	Little is known about the actual burden of disability experienced by Indigenous people, no firm data about the extent to which the use of disability support services by Indigenous people reflects their burden of disability.	R
Gunasekera,H.( 2009)	Hearing	Yes	OM management literature	Australia	To summarize best evidence for management of OM	Indigenous children with AOM should be treated with antibiotics on first visit; children with OME, and no speech and language delays, can be observed safely for 3–6 months; children with CSOM need ear cleaning & topical antibiotics.	HPs managing these children can use this evidence to make informed decisions and can discuss the pros and cons of the different management options with the child’s parents/carers	S
Burrow et al. (2009)	Hearing- strictly	No	Educational approaches	Australia	impact of hearing loss; factors contributing to hearing loss; prevention and management of otitis media and hearing loss; education strategies addressing hearing loss; and policies and policy implications for reducing hearing loss and its educational consequences.	Summaries of hearing-related screening, diagnosis, treatment, rehabilitation, language, amplification, and management issues.	Little government and policy support for research on education, social, emotional, family and community effects of Indigenous hearing loss.	R, A, S, I, Q

Otitis Media (OM); Chronic Suppurative Otitis Media (CSOM); otitis media with effusion (OME); acute otitis media (AOM); Category: (R-recognition/awareness; A-Access; S-Solutions; I-Intervention; Q-Sequelae/outcomes).

**Table 5:** Grey literature reports

**Citation**	**Design**	**Level of Evidence**	**Focus/Setting**	**Key Findings**	**Key Strategies**
Owen, L., M. Gordon, et al. (2002). Listen to us - supporting families with children with disabilities: identifying service responses that impact on the risk of family breakdown. The Family Resilience Project; Melbourne, Vic, Disability Services Division, Department of Human Services**.**	1 focus group with Aboriginal families	V	Examination of services to support families with children with disabilities/Vic	Support groups reported useful by parents; essential elements include accessibility, and common interest based on either location, Aboriginal or ethnic community bonds, or on the disabling condition of the child; living in rural/ remote areas, being members of Aboriginal communities, or of culturally and linguistically diverse communities, being on low incomes or being socially isolated, can have implications for what and how support is provided	Best practice elements - Proactive Prevention and Early Intervention; empowerment; continuity, comprehensiveness; flexibility, strengths-based, partnership, expert, culturally sensitive, promotion and protection of rights, family and child- Focused and responsive, long-term, seamless and integrated, accountability.
Couzos, S., T. Lea, et al. (2003). NACCHO ear trial and school attendance project. Deakin, ACT, Deakin, ACT: National Aboriginal Community Controlled Health Organisation, 2003.	Aboriginal community- controlled multi-center double-blind randomized controlled clinical ear trial (N = 147)	II	Impact of CSOM and treatment on school attendance of Aboriginal children/WA and QLD	High level of significant hearing disability among Indigenous Australian students; associated with learning disability, school absenteeism.	Schools should more effectively engage with health sector.
Telethon Institute for Child Health Research (2004). The health of Aboriginal children and young people: summary booklet. West Perth, WA, Telethon Institute for Child Health Research.	Survey (N = 5,289)	IV	Investigation of health of Indigenous children to inform preventative strategies promoting healthy development and well-being/WA	27% of children were limited in one or more sensory functions (vision, hearing or speech) or experienced pain.	Highlights the need for action across and beyond health sector to address the complex and inter-related factors that contribute to the increased risk of health problems amongst Aboriginal children.
Aboriginal Disability Network New South Wales. (2007). Telling it like it is: a report on community consultations with Aboriginal people with disability and their associates throughout NSW, 2004–2005. Sydney, Sydney: Aboriginal Disability Network New South Wales, 2007.	Community consultations	V	Report of community consultations throughout NSW during 2004/2005 with Aboriginal people with disability and their associates (not child- specific)/NSW	Under-representation of Aboriginal people with disability social, health, community, and disability services, related to policy and structural failures. Concerned over undiagnosed unassisted cases leading to school expulsion. High use of out-of-home care environment due to lack of awareness of support, culturally inappropriate support, fear of asking for help, lack of resources, postponing help-seeking until crisis, family and community problems.	Government and non-government service providers need to develop relationships with Aboriginal communities that are based on trust and equitable partnerships; Recognition of need for more resources to be able to meet the needs of Aboriginal owned and operated services; A focus upon early intervention programs; Prevalence studies needed Aboriginal communities nationwide
Snodgrass F., G. Groves, et al. (2007). Aboriginal students with disabilities: otitis media and conductive hearing loss. Adelaide, Adelaide: Ministerial Advisory Committee: Students with Disabilities, 2007.	case study, interviews with government, non- government, health professionals	V	Study on Aboriginal children (and families) with or at risk of developing otitis media and conductive hearing loss/SA	Perceived lack of referrals for Aboriginal children to early intervention programs; lack of relevant resources for Aboriginal families targeting ear health; ESL and access considerations; staff turnover affects relationships; Aboriginal health organizations limited in time and resources to provide community education; inconsistent information provision; difficulty accessing Aboriginal families with children under the age of 3; skilled workforce required (audiometry, teaching); school resistance to sound amplification systems.	Increase awareness and understanding amongst Aboriginal families of causes, consequences, treatments of ear/hearing health; improve preschool and school based education of hearing health and Aboriginal children's needs; service agreements between health services, including Aboriginal health teams and community groups, and education sectors
Calma T. Preventing Crime and Promoting Rights for Indigenous Young People with Cognitive Disabilities and Mental Health Issues, Australian Human Rights Commission, Sydney, March 2008	community consultations; case studies	V	Investigation of early intervention and diversionary practices aimed at preventing offending behavior in Indigenous young people with a cognitive disability and/or a mental health problem/Australia-wide	Young people with cognitive disabilities or mental health issues fall through the cracks of community social services and end up in custody; clear evidence linking low educational achievement to involvement in criminal justice system. Cultural bias in testing; inaccurate identification of Indigenous children as having cognitive disabilities; early identification and early intervention opportunities missed.	Correct assessment and diagnosis of a cognitive disability; early intervention programs targeting multiple risks; holistic and participative approach to solution development and implementation; early intervention research needed to tailor these programs to meet the needs of Indigenous peoples.

### Individual and community-related factors

#### Different conceptualisations of disability

Perceptions of Indigenous disability affect recognition and access to support and management. Reports highlighted differing views between Indigenous and non-Indigenous people’s conceptualisations of disability, impairment, and behaviours that may hinder identification, an important step towards early intervention that can improve a child’s social, language, and communication development and contribute to school readiness [32]. For example, Indigenous people with a disability may experience stigma in their communities and for some, independence may not be prioritised [32]. These views may hinder help-seeking behaviours.

While Indigenous families and communities may not perceive a child as having a disability, ‘labelling’ Indigenous people with a disability as ‘the Other’ and as a specialised field is apparent within the mainstream Australian service system [33]. Hence, appreciating these differences in meanings and understandings of disability concepts among both service providers and Indigenous peoples is important to obtaining better understandings of needs, expectations, and preferences.

Although challenges to access are a feature of disability, this is likely aggravated among Indigenous families where disclosure of a problem may lead to unwanted attention on circumstances and social situation [14]. Fear and distrust of organisations, marginalisation, and disadvantage have made some Indigenous people reluctant to access support and services [18]. Many individuals fear that their children will be taken from them [34,35]. Other factors associated with being Indigenous that impact on the experience of disability include the diversity within Indigenous communities, importance of family, community, and culture, social isolation associated with disability, and social disadvantage which alienates some families from service providers [19].

### Systems-related recognition factors

Regular hearing screening programmes in schools are essential to detect and prompt appropriate treatment for children [31]. Although screening for hearing loss is important, factors impacting on this taking place in schools include the schools’ awareness of this service, relationships developed between service providers, Indigenous perceptions of hearing loss and disability, funding, staff turnover, training and equipment, health policy, and access to health facilities [31].

#### Need for culturally appropriate screening instruments and psychometric tools

Culturally appropriate assessments are difficult to achieve as standard intellectual and social assessments have been found to be culturally-biased. D’Aprano et al. [13] found that Indigenous children were well behind non-Indigenous children using the Brigance developmental screening tool. The language, cultural relevance, and its administration method limit its use. This study has highlighted a need to adapt an appropriate instrument to guide developmental surveillance and monitoring in remote Australian Indigenous communities. It has been argued that the criteria developed for the allocation to special schooling may constitute indirect racial discrimination against Indigenous students and there is a need for assessment and evaluation by Indigenous educators of the basis on which Indigenous students are placed in special classes at school [36].

### Provider-related factors affecting recognition

Such complexities made professionals reticent to impose labels, in some cases. This can result in an underappreciated level of disability and some Indigenous students not receiving the support they need. It was discussed that if a label can facilitate additional support, a culturally appropriate professional assessment and intervention was warranted, but not otherwise [19].

#### Cultural training and workforce issues

A significant proportion of literature on hearing loss in children centres on the learning environment and teachers. Cultural considerations in behaviour, language, and conceptual differences are critical in appropriately identifying, assessing, and managing hearing loss and other disability in young Indigenous children at school [37]. Greater access to Indigenous teaching staff and Indigenous Education Workers in schools will facilitate culturally appropriate support for Indigenous students [37,38]. Professional development and cultural competence training for non-Indigenous teachers, including information about hearing loss, effective cross-cultural communication strategies, behaviour management strategies [38], and possible behavioural indicators of Indigenous children’s hearing loss [39] will contribute to Indigenous children’s school experiences as well. Ongoing training of Aboriginal community audiometrists to provide community, school, and preschool (including infants) screening programs are additional ways to address these issues [27].

#### Barriers to service access

O’Neill [32] reported that factors contributing to Indigenous use or non-use of disability support services are complex, and involve perceptions of attitudes, understandings and values regarding disability, language barriers, poor coordination between services and levels of government, workforce issues, racism among service providers, and socioeconomic disadvantage, including housing and transportation.

Access to services is often limited in remote areas [37]. Gunasekera found that rural and remote areas managed more cases per week than their urban counterparts, but had less access to specialist audiology services [40]. Urban-dwelling does not guarantee timely access to critical services, however. Although 20% of rural/remote Indigenous children wait longer to see audiology services for testing than the guideline recommended 3 months, one in eight Indigenous children nationwide wait longer than recommended for ears, nose and throat services. These results indicate that delays between presentation and treatment may be more widespread than what is generally perceived [40]. Government-funded hearing services, in particular, have been depicted as not meeting the needs of Indigenous people [37].

### Potential solutions – ways of working with Indigenous families and children

Involvement of the family in bi-cultural education was suggested as a facilitator of family engagement in primary health prevention and treatment [41]. In regards to working with health and service providers, families preferred to build a relationship over time, opportunities for discussion, and an emphasis on the abilities of the child rather than the disability [19].

Recommendations for mitigating hearing loss in schools include phonological awareness programs and hearing support services which have demonstrated improvements in literacy levels [42,43]. Use of classroom-wide sound field amplification has been found to encourage children’s interaction with teachers and peers [44]. Importantly, amplification may reduce stigma and negative attitudes of peers which have been found to inhibit hearing aide use [45,46]. Although this appears to be a rapid, cost effective part of the solution, there are no clear or enforceable standards for classroom acoustics in Australia [44].

Although not delineating specific disabilities, three articles were identified that centred on developing and maintaining relationships between allied health professionals, young Indigenous children and families, and teachers. Facilitators to successful occupational therapy access included investing and committing time to establish and maintain therapeutic relationships and using appropriate communications strategies such as gaining and giving knowledge with a respectful tone, using understandable language, and demonstrating unfamiliar tasks [47]. Additional facilitators include setting the program in culturally safe and accessible locations, such as an Indigenous health or educational setting, and involving Indigenous health workers [47-49].

### Gap in research – the family

Overall, the voices and needs of Indigenous families of children with disabilities were minimally represented in the literature identified. It was noted that teachers’ reports of behavioural issues reflective of unidentified hearing impairment, for example, can impact on the family negatively [37]. One report specifically targeting Indigenous families of children with disabilities indicated that families wanted practical support such as transport to multiple appointments and completing forms.

## Discussion

With one of the highest rates of OM in the world, it is not surprising that a preponderance of literature on Australian Indigenous child health depicts the implications of this preventable and treatable disease. Despite disability manifesting in low rates of school participation and recidivism, there are minimal data on Indigenous childhood and disability [50], as has been reported in other Indigenous populations [16]. This was apparent in the current review as well as in a recent analysis of descriptive studies describing the health, development, wellbeing of Indigenous children which found that research predominantly addressed physical health (75.1%), health determinants (27.6%), and mental health and wellbeing (2.8%) [50]. The resurgence in the literature is evident in adolescence where drug, alcohol, and incarceration manifest as evidence of marginalisation, racism, and a failure to address early childhood disabilities, many of which are treatable with early intervention [51].

Despite 53% of the Indigenous population living in major cities or inner regional areas of Australia [52], the literature predominantly focused on rural/remote-dwelling populations [50]. Likewise omitted from the extant knowledge, particularly in peer-reviewed forums, are the experiences of the Indigenous family, carers, and children and their unmet needs. This is an important focus for future research.

### Factors impacting prevention, recognition, and access

The higher rate of disability in Indigenous compared to non-Indigenous Australians is a pattern of disparity seen in many Indigenous populations throughout the world[16]. Socioeconomic disadvantage has been linked to intellectual and developmental disability, both as cause and effect, given inequitable resource allocation within social systems and subsequent contextual issues [53]. Socioeconomic disadvantage, racism, and oppression influence learning and health outcomes [16]. The absence of Indigenous Australian child disability statistics until 2006 reflects this history of dispossession, distrust, and enduring disparity. The legacy of stolen generations and a recent resurgence of government control [54] has resulted in a strong scepticism and mistrust of government organisations.

**Figure 2:**
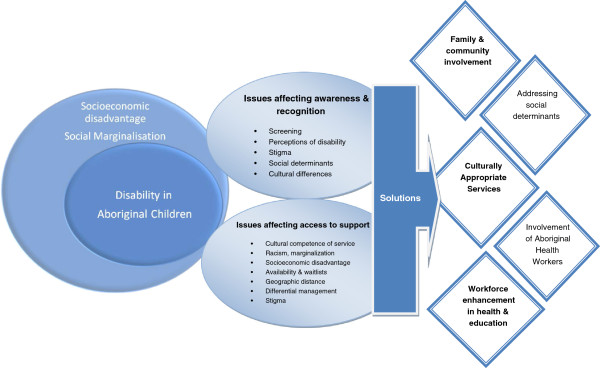
Considerations in dealing with disability in Aboriginal children.

Considerations for addressing disability in Indigenous children are depicted in Figure 2. Differences between Indigenous and non-Indigenous people’s conceptualisations of disability, impairment, and behaviours may impact on identification or diagnosis of a disability, an important step towards early intervention that can improve a child’s social, language, and communication development and contribute to school readiness [16]. As noted in the Australian literature, systematic screening for delay or impairment, particularly where English is not the first language, is vital [16].

The significant and enduring consequences of unaddressed disability were discussed and suggestions for improving awareness of cultural differences in education settings and tailoring programs to address the needs of Indigenous children provided. Johnston [55] has described education as positively associated with health through mediating pathways that exist at individual, family and community level. Johnston [55] urges clinicians to consider early childhood and school education as an important focus that requires rigorous research into interventions that address the barriers to effectiveness in implementing quality educational experiences and opportunities for Indigenous children.

There are a number of strategic initiatives to address the data shortcomings, to date, and to address factors that will maximise Indigenous participation in society. Among these is the Study of Environment on Indigenous Resilience and Child Health (SEARCH) project, a prospective longitudinal cohort study which will be a long-term resource to investigate the causes and trajectories of health and illness in Indigenous children aged 0–17 from urban and large regional centres in New South Wales, Australia, to identify potential targets for interventions to improve health. Data is being collected on OM, hearing impairment, and developmental delay [56]. Another example is the Gudaga study, a prospective descriptive longitudinal cohort study assessing the health, development, and service use of Indigenous infants and their mothers in Southwest Sydney [15]. The work of the Telethon Institute for Child Health Research is also providing a stimulus for addressing the health and well-being of Indigenous children [57].

### Strengths and limitations

This review was limited by the available published literature and therefore does not reflect the scope of the problem. Although government reports and other sources of the grey literature were reviewed for this project, sparse data reflects that many issues of childhood disability hover below the radar and only manifest when the consequences are dire and catastrophic [58]. Although a feature of disability [58], this is likely aggravated among Indigenous children where a disclosure of a problem may cast an unwanted focus on family circumstances and social situation.

Given that the needs of these populations are very unique, the outcomes of the retrieved studies may not be generalisable to the entire Australian Indigenous nor other Indigenous populations, necessarily. This review was undertaken using rigorous methods and has identified potential solutions for addressing childhood disability. This review has elucidated important contextual determinants of child health and disparities among Indigenous Australians. It also underscores the importance of shifting from a purely biomedical lens to a holistic approach to address the needs of vulnerable Indigenous children. This will require ongoing partnership and collaboration between health, education, and social service agencies.

Given the diverse study designs and inconsistent reporting, the ability of this review to decipher strength of evidence was limited. Instead, this review has provided a snapshot of the current foci of literature and approaches to describing disability in Australian Indigenous children.

### A way forward

The solutions for addressing health disparities and maximising societal participation among Indigenous Australians have been clearly identified by Indigenous people. Recognising and addressing entrenched and institutional racism is an important first step in ensuring the needs of Indigenous children are addressed [59].

This review has indicated that a great deal is still unknown about Indigenous childhood disability, yet important foci should include prevention of problems that improve health and living situations, particularly effective antenatal care. Importantly, factors that promote prevention, recognition, access, and viable solutions, as summarised in Figure 2, are warranted.

Further research must be conducted in order to provide evidence for effective and sustainable practice and policy. This is particularly the case for developing culturally appropriate interventions. Ensuring such research is led by Indigenous people using appropriate methods is a critical step in developing tailored and targeted solutions. It is critical that health and social services are accessible and acceptable to Indigenous people. Indigenous-led and controlled services are an important form of engagement. Increasing the workforce participation of Indigenous people in health, education, and social services [60] is an important step and strongly linked to addressing childhood disability and maximising participation in the education system [38].

Achieving these goals requires addressing entrenched power relationships and dismantling barriers between mainstream and Indigenous controlled services. It also will require challenging traditional professional boundaries and decreasing silos between health, education, and social services. Given the links between childhood disability and education, teachers have an essential role to play in monitoring developmental stages, thus necessitating teacher training that ensures appropriate recognition of potential disability. Greater congruency between health and education systems needs to occur, as well as other strategies to ensure children with hearing disabilities are not left behind, such as amplification systems with classrooms [61].

## Conclusions

Despite higher rates of disability than in non-Indigenous children, this review has identified the scant focus on disability in Indigenous children in the face of the burden and ramifications of not achieving educational and social milestones. Given the lack of research in this area, further investigation must be conducted. The emphasis on OM in this review reflects the hard work of a committed group of researchers, but currently, the literature fails to encapsulate the breadth and complexity of Indigenous childhood disability. Although extensive investment is apparent in downstream health problems, ensuring the foundations of health and wellbeing through early intervention is critical in decreasing health inequity.

## Abbreviations

WA: Western Australia; CINAHL: Cumulative Index to Nursing and Allied Health Literature; APAIS-ATSIS: Indigenous Australia, Australian Public Affairs Information Service-Aboriginal and Torres Strait Islander; ATSIHealth: Aboriginal and Torres Strait Islander Health; ERIC: Education Resources Information Center; MeSH: Medical Subject Headings; STROBE: STrengthening the Reporting of OBservational studies in Epidemiology; TREND: Transparent Reporting of Evaluations with Nonrandomized Designs; OM: Otitis Media; SEARCH: Study of Environment on Indigenous Resilience and Child Health.

## Competing interests

The author(s) declare that they have no competing interests.

## Authors’ contributions

MD contributed to conception and design, acquisition and analysis of data, and manuscript drafting and revision. PMD contributed to conception and design, acquisition and analysis of data, and manuscript drafting. TD acquired and analysed data and revised the manuscript. SJM contributed to data analysis and manuscript drafting. PD contributed to study conception, design, and review. PA contributed to conception, design, and manuscript revision. JD contributed to study conception and review. FV contributed to study conception and review. All authors read and approved the manuscript.
